# Clinical applications of artificial intelligence and machine learning in neurocardiology: a comprehensive review

**DOI:** 10.3389/fcvm.2025.1525966

**Published:** 2025-04-03

**Authors:** Jade Basem, Racheed Mani, Scott Sun, Kevin Gilotra, Neda Dianati-Maleki, Reza Dashti

**Affiliations:** ^1^ Renaissance School of Medicine at Stony Brook University, Stony Brook, NY, United States; ^2^ Department of Neurology, Stony Brook University Hospital, Stony Brook, NY, United States; ^3^ Department of Medicine, Division of Cardiovascular Medicine, Stony Brook University Hospital, Stony Brook, NY, United States; ^4^ Department of Neurosurgery, Stony Brook University Hospital, Stony Brook, NY, United States

**Keywords:** artificial intelligence, machine learning, deep learning, cerebrovascular, ischemic stroke, cardiovascular, neurocardiology

## Abstract

Neurocardiology is an evolving field focusing on the interplay between the nervous system and cardiovascular system that can be used to describe and understand many pathologies. Acute ischemic stroke can be understood through this framework of an interconnected, reciprocal relationship such that ischemic stroke occurs secondary to cardiac pathology (the Heart-Brain axis), and cardiac injury secondary to various neurological disease processes (the Brain-Heart axis). The timely assessment, diagnosis, and subsequent management of cerebrovascular and cardiac diseases is an essential part of bettering patient outcomes and the progression of medicine. Artificial intelligence (AI) and machine learning (ML) are robust areas of research that can aid diagnostic accuracy and clinical decision making to better understand and manage the disease of neurocardiology. In this review, we identify some of the widely utilized and upcoming AI/ML algorithms for some of the most common cardiac sources of stroke, strokes of undetermined etiology, and cardiac disease secondary to stroke. We found numerous highly accurate and efficient AI/ML products that, when integrated, provided improved efficacy for disease prediction, identification, prognosis, and management within the sphere of stroke and neurocardiology. In the focus of cryptogenic strokes, there is promising research elucidating likely underlying cardiac causes and thus, improved treatment options and secondary stroke prevention. While many algorithms still require a larger knowledge base or manual algorithmic training, AI/ML in neurocardiology has the potential to provide more comprehensive healthcare treatment, increase access to equitable healthcare, and improve patient outcomes. Our review shows an evident interest and exciting new frontier for neurocardiology with artificial intelligence and machine learning.

## 1 Introduction

### 1.1 Neurocardiology

**Figure 1:**
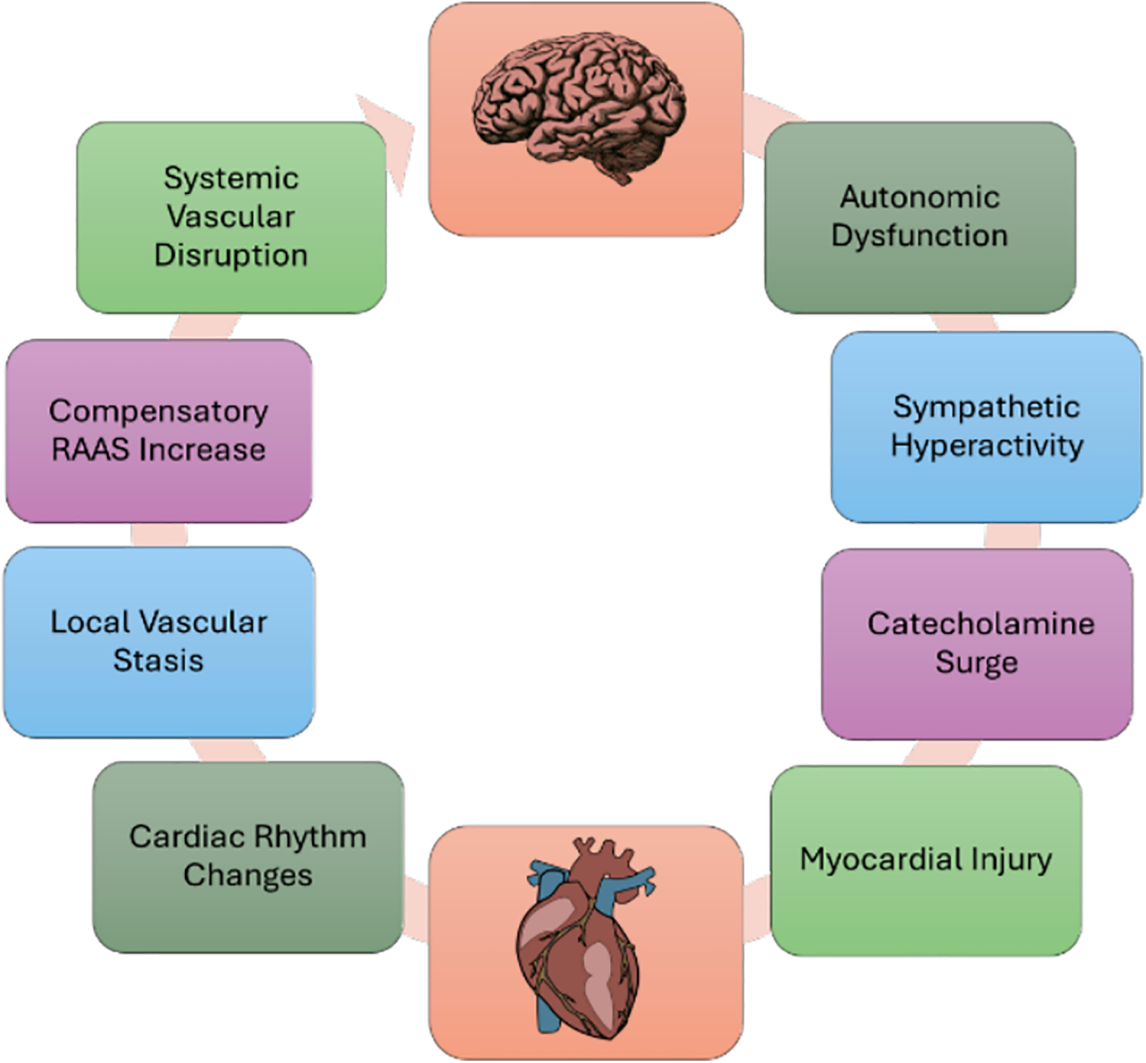
Basics of neurocardiology.

Neurocardiology is an evolving field with particular focus on the interplay between the nervous and cardiovascular systems (Figure 1). Most frequent clinical scenarios include hemorrhagic and ischemic stroke secondary to cardiac pathology (the Heart-Brain axis), and cardiac injury secondary to various neurological disease processes (the Brain-Heart axis) (1–4). This framework suggests an interconnected, reciprocal relationship between the two systems, whereby cardiac dysfunction can result in various scenarios of ischemic or hemorrhagic stroke, and conversely, intracranial disorders can impact cardiac function and physiology (5–10). Various biological mechanisms have been implicated in this reciprocal relationship of pathophysiology, ranging from autonomic dysregulation to systemic inflammation affecting both systems (5–7, 11).

### 1.2 The heart-brain axis: cerebrovascular disease secondary to cardiac pathology

The heart-brain axis is of significant relevance to the ischemic stroke population (12–14). Up to 25% of adults will suffer from at least one stroke in their lifetime and it represents the second-leading cause of death worldwide and the third-leading cause of disability (15–19). These numbers have consistently increased over the past 20 years, with stroke recurrence rates reportedly as high as 54% in Acute Ischemic Stroke (AIS) outcomes (16, 20–24). Functional outcomes, stroke recurrence, and secondary stroke prevention vary widely depending on stroke etiology (15, 25). Based on the recommendations of the 2021 American Heart Association/American Stroke Association Guidelines, the Trial of ORG 10172 in Acute Stroke Treatment (TOAST) classification is widely used to classify the etiology of AIS (Table 1) (26, 27).

**Table 1:** The trial of Org 10172 in acute stroke treatment (TOAST) classifications.

1. Large-Artery Atherosclerosis (embolus/thrombosis) 2. Cardioembolism (high-risk/medium risk) 3. Small-Vessel Occlusion (lacune) 4. Stroke of Other Determined Etiology 5. Stroke of Undetermined Etiology (two or more identified, negative evaluation, incomplete evaluation)

Within the TOAST classification system, stroke secondary to emboli of cardiac origin (cardioembolism) represent the largest subgroup of stroke patients at 30%, with embolic strokes of undetermined source (ESUS) at 25% (26, 28). Moreover, various reports suggest that ESUS, traditionally categorized as cryptogenic strokes, can be often diagnosed as cardiac in origin with comprehensive cardiac workups (21, 28–36).

Cryptogenic stroke represents a category of stroke not attributable to any of the other four major TOAST subtypes (Table 1), accounting for approximately 20%–30% of all ischemic strokes. Recent studies have introduced the concept of ESUS as a specific subtype within cryptogenic strokes, aimed at identifying patients who may benefit from enhanced imaging studies or treatment such as anticoagulation even in the absence of documented atrial fibrillation (AF) (37, 38). Rarely, hemorrhagic stroke can also be caused cardiac dysfunction, such as in infective endocarditis and myocardial infarction (39–42).

### 1.3 The brain-heart axis: cardiac disease secondary to cerebrovascular pathology

The bidirectional nature and interconnected pathophysiology of the nervous and cardiovascular systems are also evident by the high prevalence of cardiac abnormalities following different types of ischemic stroke, giving credence to the notion of a “brain-heart axis” (2–5, 7, 9, 12–14, 43, 44). The pathologies of the brain-heart axis most notably include neurogenic stress cardiomyopathy (including Takotsubo cardiomyopathy), arrhythmias, and acute myocardial injury, and can be seen as early as 24 h following stroke (5, 7, 14, 45). Moreover, post-stroke cardiac injury is associated with higher mortality rates, more adverse long-term outcomes, and increased rates of stroke recurrence (14, 20, 26, 43, 46–49).

**Figure 2:**
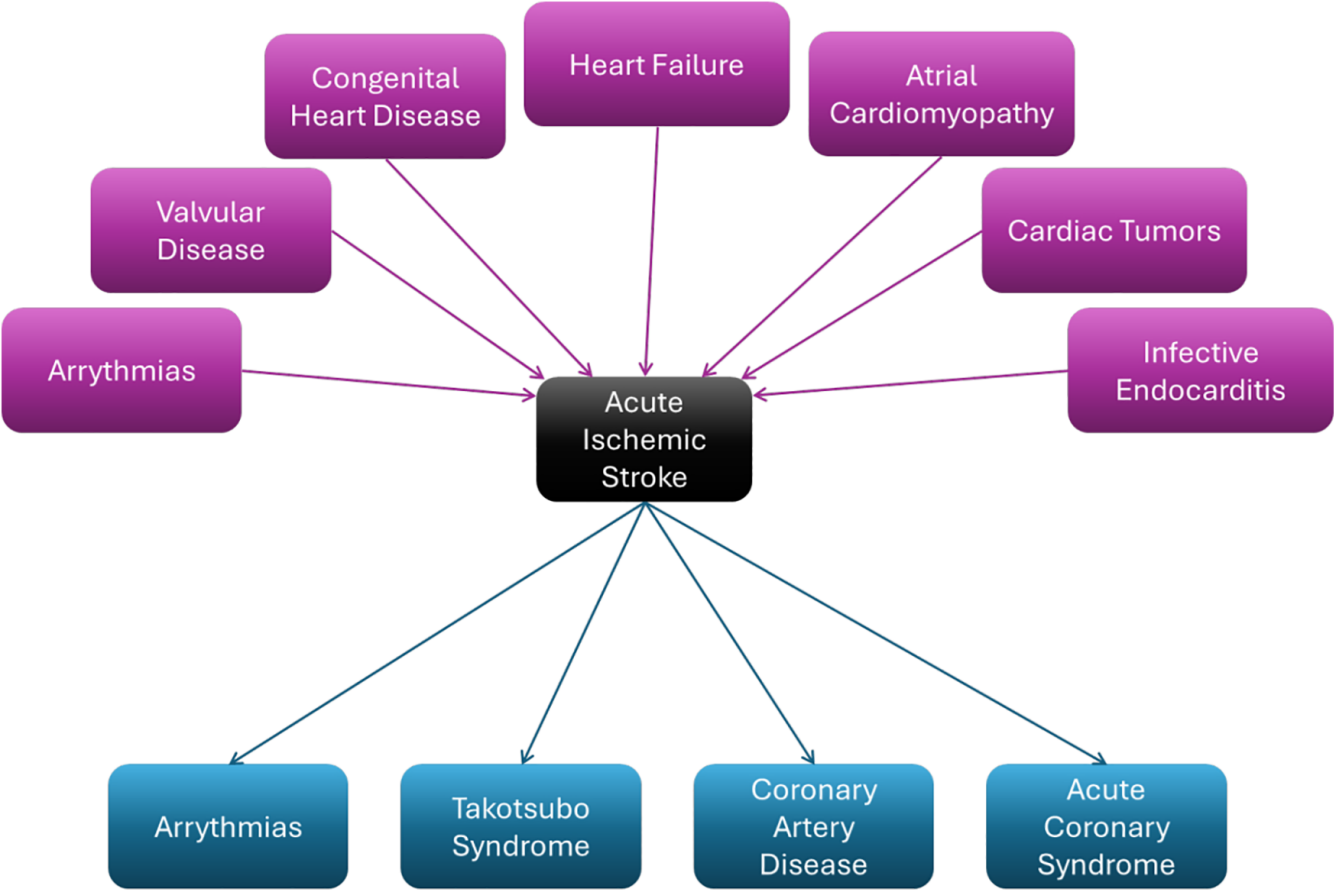
Heart-brain and brain-heart axis of acute ischemic stroke.

The mechanisms behind cardiac dysfunction after central nervous system (CNS) injury may be rooted in a maladaptive stress response causing acute injury and prolonged structural changes (Figure 2). Autonomic dysfunction is among the most significant disease processes underlying the heart-brain axis. Heart rate variability can be used to assess both general autonomic regulation and cardiac autonomic stability (7, 13, 50, 51). Heart rate, heart rate variability, and ratio of high frequency to low frequency bands can indicate the levels of sympathetic vs. parasympathetic activity and may therefore be used as a predictor for outcomes and mortality (7, 52–56).

Within the central nervous system, the deleterious effects of an infarct on specific brain structures can alter the interpretation of cardiac response/stress. For instance, the insular cortex, supplied by the middle cerebral artery (MCA) and commonly injured in AIS, is associated with cardiorespiratory interoception and autonomic responses (12, 57, 58). Furthermore, its connection to structures such as the amygdala also demonstrates its role in the response to emotional stress and to a patient's interpretation of cardiac changes such as heartbeat variability (12, 58, 59). Emotional stress is also implicated in “mental stress-induced myocardial infarctions” with alterations in catecholamine release also contributing to cardiac alterations and microinfarcts (60–62).

In addition to pathological changes within the brain, these changes in structure and response to stress also manifest downstream in the heart, through direct electrical cardiac alterations, endothelial disruptions, and cardiac remodeling. One such example is Takotsubo syndrome, which manifests in part due to emotional stress, but also commonly occurs in patients with co-existing neurological disorders, with a prevalence ranging from 6% to 17% (63–65). Takutsubo syndrome has conferred worse functional outcomes and increased in-hospital and long-term mortality rates for patients with neurological disease (64, 65). Similarly, the suspected disease processes in this syndrome consist of an aberrant autonomic stress response secondary to injury to brain structures associated with regulating autonomic function, including the amygdala, hippocampi, the right putamen, and the left superior temporal pole. This leads to perturbations in connectivity among these structures, a dysregulated sympathetic response, and increased catecholamine release leading to myocardial insult and cardiac dysfunction (63, 64, 66–69).

### 1.4 The role of artificial intelligence in clinical applications of neurocardiology

The need for timely assessment, diagnosis, and subsequent intervention in this patient population is essential to optimize patient outcomes. The evolution of artificial intelligence (AI) and machine learning (ML) has provided a potentially groundbreaking avenue to develop useful clinical tools to optimize the detection, prognosis, and treatment of cerebrovascular diseases (70–72).

In brief, AI entails the use of a machine to simulate human intelligence to solve tasks (73). Within AI is machine learning (ML), which encompasses the iterative training of machines using large volumes of data to detect patterns and generate problem-solving models which can be applied to future data sets (73). Deep learning (DL) is a subclassification of ML which entails the use of interconnected neural networks to simulate the process of learning in humans (73).

Utilizing trained algorithms, AI often has high accuracy for diagnosis, prognosis of disease progression, and treatment suggestions, while also integrating historical data and personalized patient information (74). Several AI programs have been approved by the Food and Drug Administration (FDA) and have been implemented in many hospital systems worldwide for the detection of cerebrovascular accidents (CVAs), utilizing imaging modalities such as MRI, CT scans, computed tomography angiography (CTA) and computed tomography perfusion (CTP) scans, and to assess the cerebral vasculature and blood flow (70, 75, 76).

We have previously reviewed the growing applicability and implementation of AI/ML algorithms in the diagnosis, management, and prognostication of cerebrovascular diseases (70). Most pertinent to neurocardiology is ischemic stroke, for which these algorithms have shown a high degree of diagnostic efficacy, utilizing CTAs to detect large vessel occlusions (LVOs) and in using CTP scans to estimate perfusion mismatch and core infarct volumes (70). Moreover, AI/ML algorithms have also proven to be highly effective.

The prognostication for stroke patients can be performed using grading systems such as the Alberta Stroke Program Computed Tomography Score (ASPECTS). This score is used to predict stroke severity and clinical outcomes based on the anatomical distribution of stroke and the division of the MCA affected in cases of LVO (70). For what ordinarily is a cumbersome process of tabulation for humans, AI/ML provides an expedited means of tabulating these scores, which is crucial in the acute setting of ischemic stroke in which time is of the essence (70). These algorithms have either outperformed or matched the accuracy of human radiologists, are now increasingly implemented into routine clinical practice for ischemic stroke. Commercial platforms such as RapidAI and Brainomix are now regularly used as a supplementary tool for clinical decision-making for stroke teams (70).

The use of AI/ML applications in stroke assessment has therefore already shown clinical significance due to their ability to rapidly combine and analyze large volumes of patient input information when compared to manual computation or assessment (77–81). While this technology yields its highest accuracy in the diagnosis of cardioembolic strokes, its accuracy for the diagnosis of ESUS is also improving with further iterations of AI/ML algorithms (80). AI/ML has also been used as a predictive tool for cerebrovascular and cardiovascular disease. This is of particular importance from the perspective of neurocardiology, in which modifiable, preventable comorbidities such as atherosclerosis are heavily interconnected and implicated in the pathophysiology of both systems.

In addition to their utility for clinical decision making, AI algorithms are also efficient analytical tools, can reduce human error, and can identify trends otherwise missed by the human eye or standard calculations (75, 80, 82). This is particularly essential in acute disease processes such as stroke, where accuracy and speed can drastically affect patient outcomes. The implementation of automation can also reduce physician fatigue when used correctly for personalized care and decision making (83, 84). AI may also be able to help patients receive more equitable stroke management by providing accessible and personalized care options to reduce geographical, socioeconomic, and ethnic health disparities (82, 85, 86). However, the literature consistently notes that the benefits of AI applications are inherently limited by the ability to standardize its implementation and train clinicians to effectively use it (82).

Therefore, it is imperative for clinicians to be familiar with current and future stroke evaluation modalities in a rapidly evolving, technological landscape. In this review, we will evaluate the role of AI and ML in the heart-brain relationship in stroke, focusing both on the most common cardiac causes of stroke and the most common post-stroke cardiac complications.

## 2 The heart-brain axis: AI/ML applications in cardiac sources of stroke

### 2.1 Atrial fibrillation

According to the American Heart Association (AHA)/American Stroke Association's 2021 Guideline for the Prevention of Stroke in Patients with Stroke and Transient Ischemic Attack, atrial fibrillation (AF) is the most common arrhythmia in adults, and the leading cause of acute ischemic stroke (75). Initially diagnosed by electrocardiogram (EKG), AF confers an increased risk of future stroke, with the need for anticoagulation to mitigate this stroke risk determined by the CHA_2_DS_2_-VASc risk score (87). Despite up to 40% of AF patients being asymptomatic, these patients are at a higher risk of mortality than even symptomatic patients (87). This is likely due to treatment nonadherence and insidious disease progression while asymptomatic. One AF subtype, paroxysmal AF, has an intermittent and unpredictable presentation, requiring longer diagnostic monitoring, and is often missed during hospital admissions during stroke. Certain risk models, such as the CHARGE-AF score, is often implemented to identify high risk patients based on clinical factors without the need for EKG data (88). While AF is the most common cause of the cardioembolic TOAST subcategory, it may also represent up to 15% of all cryptogenic strokes (26, 32, 79).

#### 2.1.1 Diagnostic electrocardiography

**Figure 3:**
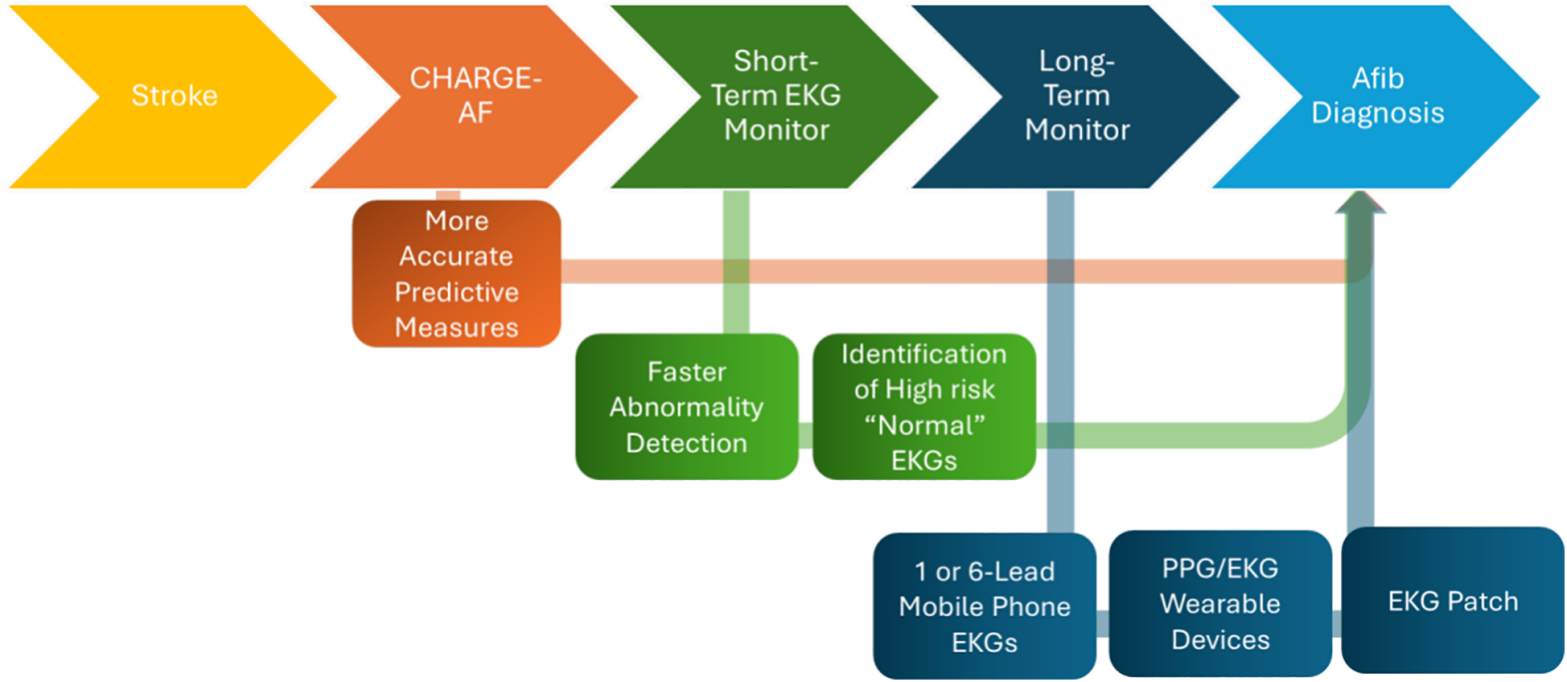
Implementation of AI for in-hospital EKG evaluation and diagnosis of atrial fibrillation.

AF diagnosis with EKG is one of the most widely studied implementations of AI in AIS/neurocardiology diagnosis and identification to stroke risk stratification and treatment options (Figure 3). This includes several FDA-approved programs such as Cardiomatics and AccurKardia (89, 90). Certain AI/ML models have been shown to outperform validated tools such as CHA_2_DS_2_-VASc in stroke prediction (91, 92).

While normal sinus rhythm on initial EKG may point clinicians away from a diagnosis of AF, Attia et al. recently used over 20 years of EKG data from the Mayo Clinic to assess the ability of AI to identify AF from a normal sinus rhythm on EKG (93). This study demonstrated area under the curve (AUC) of 0.87 for correctly diagnosing AF among patients who had previously recorded AF rhythms. However, this AUC increased to 0.90 when predicting AF in undiagnosed patients, notably if EKG was obtained up to 31 days prior to the first EKG-recorded AF episode (93). This AI apparatus was then compared to the validated CHARGE-AF score for AF risk prediction and performed with a similar degree of accuracy (94). These measures have also been used in the assessment and diagnosis of AF in the setting of cryptogenic stroke (95). Additionally, Weil et al. investigated the predictive value of an AI-generated EKG score of AF risk, noting that a higher AI-EKG-AF score correlated with the subsequent development of cerebral infarcts on MRI, therefore demonstrating the value of such AI-algorithms in predicting cerebrovascular disease even with EKG data alone (96).

Based on these findings, Raghunath et al. subsequently developed their own deep neural network (DNN), while also including a 1-year predictive factor for future-onset AF for a sample of 430,000 patients over 30 years. This DNN demonstrated a final AUC of 0.85 and a sensitivity of 69% in a simulated deployment scenario (97). The authors analyzed their study cohort and noted that 62% of patients with predicted AF subsequently suffered from stroke within 5 years of the study, a greater accuracy than currently approved protocols (97). Kim et al. also followed a similar 12-lead EKG model mainly using ResNet blocks, but also incorporated an attention analysis for the model using Grad-CAM with convoluted neural network (CNN) feature map gradients (98). The authors noted that sinus rhythm EKGs with prolonged QRS compared to a specific point between the T and P waves correlated with an AI-diagnosis of AF (98).

Most of these studies also found that monitoring close to an AF event provided better results and eliminated the indication for long-term monitoring in this population (97, 98). New models are continually being created to improve diagnostic efficacy and risk prediction (99, 100).

##### 2.1.1.1 Mobile electrocardiography

Mobile EKGs (mEKGs) are an increasingly popular mode of cardiac evaluation through commonly worn devices with FDA approval, such as Apple Watch single-lead EKGs, and approved monitoring tools such as the KardiaMobile with 6-lead EKGs (101–106). These novel assessment tools allow for close monitoring at the outpatient, community level, which may improve the rate of detection and therefore allow for early preventative measures for stroke (106–108). Screening measures with clinicians using automated AI with mEKGs have yielded sensitivities as high as 98.5% and specificities up to 91.4% (108). The use of AI with this mEKG modality has also provided a means of easing clinician workload and overcoming restrictions during the height of the COVID-19 pandemic (106, 109). Furthermore, mEKGs may also be vital in the diagnosis of paroxysmal, asymptomatic AF through providing a means of extended cardiac monitoring.

Raghunath et al. have also utilized KardiaMobile data using over 260,000 mEKG recordings to test their own AI and DL algorithm utilizing a combination of CNN-based ResNet layers, gated recurrent unit (GRU), and standardized dense neural network layers that built upon Attia et al.'s 2019 model using classic 12-lead EKGs (93, 104). They assessed for paroxysmal AF events, noting a high positive predictive value when the algorithm was trained within 2 days of an event (104). This ability to accurately predict future AF events could drastically change medical management in asymptomatic patients.

For an ischemic stroke population, in-hospital use of this technology may capture an additional AF event and provide more accurate diagnosis during admission. When comparing this data to 12-lead EKG AI models or the CHA_2_DS_2_-VASc risk score, these technologies have tended to outperform risk measure scales but have demonstrated reduced accuracy than 12-lead EKGs even with similar algorithm priorities (103, 104). Regardless, this technology provides an accessible and reliable alternative for monitoring patients at high-risk for AF and subsequent stroke.

##### 2.1.1.2 Electrocardiography patch

Continuous EKG monitoring may also be conducted through a patch, such as the FDA approved iRhythm Zio and BioTel MCOT Patch (106, 110). The clinical use of these AI algorithms with immediate monitoring has demonstrated high accuracy in diagnosis of AF and has led to earlier initiation anticoagulation to mitigate the risk of thrombus formation and subsequent embolization to the brain (110, 111). Various studies have also demonstrated that the patch yielded slightly improved rates of detection compared to classic Holter monitors which have limited long-term ambulatory functional use (112–116). Therefore, there is a growing role for AI not just in the emergency setting, but also in providing an invaluable means of cardiac monitoring at home or outpatient for paroxysmal AF (117).

#### 2.1.2 Diagnostic photoplethysmography

Photoplethysmography (PPGs) are light-based sensors for blood flow measurement utilized both in the inpatient and outpatient settings in wearable devices such as smartwatches. The WATCH AF trial was among the first studies demonstrating the feasibility of AF diagnosis and observation in PPG-enabled devices, citing an accuracy of 96.1% (118). A recent systematic literature review found 24 cohort studies mostly assessing AF detected by smartwatches showing its significant growth and popularity in recent years, with several studies pointing to the easy implantation of these tools (119–122). PPG technology may also be used as a mobile phone application, such as with FibriCheck and SMARTBEATS, applications which have yielded high diagnostic accuracy, with sensitivity and specificity rates of >95% when compared to the diagnostic ability of single-lead EKGs (123–125).

### 2.2 Valvular disease

Valvular heart disease (VHD), a major cause of morbidity and mortality worldwide, has been recognized as an underlying risk factor for ischemic stroke. Both mitral and aortic valve stenosis have been associated with cardioembolic events, and the prevalence of VHD has been steadily increasing with an aging population (126). AI platforms may potentially enhance diagnostic capabilities, ease workflow, and potentially improve the outcomes of the patient with VHD and ischemic stroke. The role of AI in diagnosing valvular heart disease has been explored through assessing EKGs, phonocardiograms, and echocardiograms.

#### 2.2.1 Diagnostic electrocardiography

AI-enabled electrocardiogram models have been developed, utilizing CNNs to assist in the early detection of significant aortic stenosis (AS) (127). Using a sample of over 258,000 of EKGs to train, validate and test the model, researchers at Mayo Clinic, Rochester developed an AI-EKG system capable of predicting moderate to severe AS with impressive sensitivity, specificity and accuracy (127).

#### 2.2.2 Diagnostic transthoracic echocardiography

Since echocardiography is the primary modality for diagnosis and evaluation of VHD, the primary focus of AI research in valvular heart disease remains on the review and interpretation of echocardiograms. AI can assist in the segmentation of valves and cardiac structures for automated analysis. Using image recognition algorithms, aortic and mitral valve disease states have been directly detected from the images themselves. Several AI-enabled algorithms have been developed to diagnosis mitral valvular disease via quantitative analysis of 3-dimensional (3-D) echocardiography. These include Mitral Valve Quantification (Philips Medical Imaging, Andover, MA), eSie Valve (Siemens Healthcare, Mountain View, CA, USA), Mitral Valve Navigator (Philips Medical Systems), Auto Valve Analysis (Siemens; California, USA), and eSie PISA Volume Analysis (Siemens Medical Solutions USA, Inc., Mountain View, CA) (128). Measurements obtained during echocardiographic valvular assessment have been integrated with other clinical data to identify novel aortic valve disease subgroups and describe new predictors of aortic valve disease progression (129).

Similarly, another FDA-approved AI-apparatus is iCardio.ai, which has been cleared for use in the detection of AS This apparatus was developed through training and testing CNNs to use two-dimensional (2D) TTE to diagnose AS, with an AUC of 0.96 (130). Since severe aortic stenosis is a known risk factor for ischemic stroke, screening of at-risk populations with timely and efficient AI-enabled tools can be valuable.

#### 2.2.3 Cardiac magnetic resonance imaging

AI applications can provide a more rapid analysis of cardiac MRI data, automated and quantitative evaluations of the valvular structures, and a proper appraisal of patient prognosis in the setting of disease (131, 132). This information is essential in gauging disease severity and stroke risk.

##### 2.2.3.1 MRI segmentation

A study by Alabed et al. demonstrated that DL algorithms for cardiac MRI segmentation and measurement have similar or better efficiency as compared to manual measurements (133). In their multicenter dataset, intraclass correlation coefficients (ICCs) ranged from 0.94 to 0.99 for left ventricular (LV) and right ventricular (RV) volumes respectively, with similarly high ICCs for ejection fractions (LV: 0.93, RV: 0.94), and ventricular mass (LV: 0.95, RV: 0.92). Comparing dice analysis of their internal and external test cohorts showed significant concordance between manual and AI measurements (133).

These findings are further supported by Bai et al. with their fully convolutional network (FCN) based cardiovascular resonance (CMR) image analysis (134). The team found that the performance of the FCN-based automated analysis was on par with human inter-observer variability for metrics such as LV and RV end-diastolic volume (EDV) with a mean absolute difference of 6.1 ± 5.3 ml and 8.5 ± 7.1 ml, respectively. MRI has also been successfully quantified using AI algorithms based on the proximal isovelocity surface area (PISA) method, with phenotyping studies demonstrating promising results in the detection and quantification of AS and mitral regurgitation (MR) (135–137).

These results support the notion that automated methods based on AI-modeling and proper dataset training can improve performance and predictability of multiple modalities.

##### 2.2.3.2 MRI flow imaging

AI models have also been integrated into difficult workflows such as four-dimensional (4D) flow magnetic resonance imaging for capturing temporal changes of blood flow patterns. Aortic regurgitation onset and severity can be measured with 4D flow MRI, however, the metrics obtained are extremely sensitive to spatial resolution. Long et al. used deep-learning upscaling the increase resolution by a factor of four. Building upon the existing 4DFlowNet model, they devised three architectures 4DFlowNet-Res, 4DFlowNet-Dense, and 4DFlowNet-CSP, all of which outperformed the baseline model in root mean square error (RMSE), structural similarity index (SSIM), and relative speed error (RE) showing how AI-integration can improve existing assessment workflows (138).

#### 2.2.4 Diagnostic phonocardiography

DL algorithms have also been utilized in digital stethoscope platforms capable of automated cardiac auscultation and murmur detection. AI-assisted cardiac auscultation in practice refers to the auto-interpretation of phonocardiograms, within the domain of signal processing. Various algorithms can diagnose severe AS with sensitivity and specificity of over 90% (139). Chorba et al. developed and tested a deep CNN model that classifies phonocardiograms in 3 categories: heart murmur, no murmur, or inadequate signal. To determine the accuracy of the signal quality and murmur detection performance, the algorithm output was compared with annotations from three cardiologists. This algorithm yielded a sensitivity and specificity for detecting murmurs of 76.3% and 91.4%, respectively. The study also used the gold standard echocardiogram to detect clinically significant (deemed as moderate or greater) AS and MR, with moderate success. Detection of AS was 93.2% sensitive and 86.0% specific, and detection of MR was 66.2% sensitive and 94.6% specific (139).

AI-platforms can be useful in screening for valvular heart disease through auscultation. One such AI-apparatus recently approved by the FDA is the Eko Murmur Analysis Software (EMAS), a “smart” stethoscope which can detect and characterize even asymptomatic murmurs in both pediatric and adult patients (140). This valuable tool which can be used on a daily basis during physical exams, can assist with early diagnosis of valvular heart disease such as aortic and mitral valve stenosis which are among known risk factors for ischemic stroke.

#### 2.2.5 Stroke prediction

Artificial intelligence and machine learning have emerged as powerful tools to aid clinicians in predicting stroke and related outcomes. These models have demonstrated equal or superior performance to traditional statistical methods, prompting further investigation into the potential for AI/ML integration into risk assessment and prognostic prediction for stroke across various cardiovascular conditions.

Zhou et al. utilized machine learning algorithms to predict stroke and mortality in patients with mitral regurgitation (141). They found that a gradient boosting machine (GBM) method significantly outperformed many traditional statistical methods in predicting adverse outcomes of TIA/stroke and all-cause mortality. For prediction of TIA/stroke, the GBM model achieved an AUC of 0.8084 in comparison to 0.4128 for logistic regression (LR), 0.5528 for decision tree (DT), 0.7202 for random forest (RF), 0.7429 for support vector machine (SVM), and 0.7533 for artificial neural network (ANN). For prediction of all-cause mortality, the GBM model achieved an area under the receiver operating characteristic curve (AUC) of 0.7962 in comparison to 0.4063 for LR, 0.5490 for DT, 0.7132 for RF, 0.7354 for SVM, and 0.7702 for ANN (141).

Extending this research, Rauf et al. applied a similar GBM model to patients with mitral stenosis with atrial flutter (142). Their results further confirm the GBM model's efficacy, with an AUC of 0.8037 for TIA/stroke risk prediction and 0.8388 for all-cause mortality risk prediction, significantly outperforming traditional statistical methods with AUC ranging from 0.4392 to 0.6976 for TIA/stroke and 0.4719 to 0.6174 for all-cause mortality (142).

In context of AS, Shimoni et al. trained a random survival forest (RSF) model using data from an AS registry to assess prognostic characteristics with an AUC of 0.83 (0.80–0.86) for 1-year outcome and 0.83 (0.81–0.84) for 5-year outcome (143).

### 2.3 Congenital heart disease

Congenital heart disease (CHD) encompasses structural cardiac abnormalities manifesting from birth, with significant impacts on lifetime health such as increased stroke risk at a younger age (144, 145). The presence of a patent foramen ovale (PFO) is among the most common congenital abnormalities seen in 25% of the population. This is characterized by a persistent aperture between the right and left atria of the heart. Atrial septal defect (ASD) is another cause of intra-cardiac shunting and accounts for 10%–15% of all CHD. Both PFO and ASD create a conduit for paradoxical thromboembolisms to the brain, placing CHD among the most common cardiac etiologies of AIS (146–149).

#### 2.3.1 Stroke prediction

Several predictive scales have been developed to assess the risk associated with congenital abnormalities, particularly PFO, and their subsequent risk for AIS. The Risk of Paradoxical Embolism (RoPE) score and the PFO-associated Stroke Causal Likelihood (PASCAL) risk stratification system are the most widely utilized risk scales (148, 150). The RoPE score consists of age, hypertension, diabetes mellitus, smoking history, previous stroke, or transient ischemic attack (TIA), and neuroimaging findings to estimate the likelihood of a stroke occurring due to a PFO. Higher RoPE scores indicate a greater probability of a stroke occurring due to a pathologic PFO, but conversely, are associated with a lower risk of recurrent stroke (148, 149, 151). The PASCAL risk stratification system categorizes patients into subgroups based on the presence of high-risk PFO features, such as a large shunt or atrial septal aneurysm, in conjunction with the RoPE score (148, 149).

There have been several studies utilizing ML algorithms for stroke risk in patients with documented CHD. Bai et al. constructed a RF ML algorithm in which they established the relationship between cryptogenic stroke and predictor variables, including morphologic and functional characteristics of PFO, as detected by transthoracic and transesophageal echocardiogram (TTE/TEE) (146). Traditional algorithmic approaches such as SVM and ANN have been considered. However, the high dependence on database accuracy and time efficiency rendered these approaches suboptimal.

#### 2.3.2 Recurrent stroke prediction

Luo et al. constructed their random forest survival (RFS) model under supervised machine learning and used random forest variable importance (VIMP) to select variables such as fasting blood glucose, thickness of interventricular septum, ratio of mitral peak early (E) to late (A) diastolic filling velocity, left ventricular end-systolic dimension, body mass index, systolic blood pressure, and thickness of the posterior wall from a population of patients with post-closure PFO diagnosed by TTE/TEE (148). Compared to the concordance index (C-index) of 0.54 in the traditional Cox proportional hazard regression model, the RFS model had a C-index of 0.87. RFS analysis supported traditional risk factors and identified the ratio of mitral peak early to late diastolic filling velocity and the thickness of the interventricular or posterior wall in post-closure PFO patients as predictive values to prevent stroke recurrence.

In another study in adult patients with CHD by Chiriac et al., two ML models (RegCOX and XGBoost) were constructed as prediction models for stroke and systemic embolism (SSE) (144). Both models were compared against and significantly outperformed the traditional CHA_2_DS_2_-VASC score among the ACHD population with AUC at 5 years of 0.80 for RegCOX, 0.79 for extreme gradient boosting (XGBoost), and 0.74 for CHA_2_DS_2_-VASC. Both ML models included the CHA_2_DS_2_-VASC score as part of their regression in addition to history of cerebrovascular disease, ASD, and the Charlson comorbidity index as the other high-risk variables.

PFO size has been implicated with increased mortality rates in AIS patients and clinical studies have shown varying efficacy for PFO closure vs. medical therapy for secondary stroke prevention with high-risk PFO. Factors such as age and size of PFO have been considered in past clinical trials to support the closure of high-risk PFO and increasing data has supported PFO closure to significantly reduce the risk of stroke or TIA. The analysis by RFS in Luo et al. supported continued targeting of high-risk groups even post-PFO closure (148). ASD, as identified by the RegCOX and XGBoost models of Chiriac et al. has been identified as a significant risk factor for late morbidity and mortality with normal survival reported following early ASD closure. The PASCAL risk stratification has shown in a recent meta-analysis that high-risk PFO features and RoPE score together yield strong predictive value of recurrent ischemic stroke in closure with a 90% relative risk reduction in the PROBABLE group, 62% in the POSSIBLE group, and no significant difference in the UNLIKELY group (149). While the closure of PFO holds promise as a therapeutic intervention for recurrent ischemic stroke, challenges and questions regarding optimal timing of PFO closure, prophylactic PFO closure in adults with CHD, long-term durability of closure devices, potential risks and patient safety factors are still yet to be studied (149).

### 2.4 Heart failure

Heart failure (HF) can manifest as a sequela of progression of any cardiovascular pathology. HF affects at least 26 million people globally with a high morbidity and mortality, and imposes a significant medical and financial burden on patients and hospitals (152). HF is among the leading causes of hospitalization in the U.S. alone, with at least 1 million admissions with HF as a primary diagnosis annually (152, 153). The prevalence of HF is anticipated to grow further due to increasing life expectancy coupled with increased incidence in cardiovascular risk factors such as obesity, diabetes, hyperlipidemia, and myocardial infarction (MI) (154). Specific to neurocardiology, HF confers a two to three-fold increase in the risk of ischemic stroke, largely due to the risks of cerebral hypoperfusion and thromboembolism (155).

The diagnosis and management of HF entails assessing myocardial pump function through various imaging modalities. Echocardiography remains the cornerstone in assessing cardiac function. Decreased cardiac pump function occurs due to a variety of underlying pathophysiologies and is diagnosed by two-dimensional (2D) echocardiography or myocardial strain analysis by Speckle-tracking echocardiography (STE). Decreased cardiac function leads to impairment of cardiac output (CO) and presents HF symptoms. This state of decreased CO in turn can cause cerebral hypoperfusion and is associated with increased risk of ischemic stroke. Moreover, stasis of blood in dysfunctional cardiac chambers increases the risk of thrombus formation which is a known risk factor for embolic strokes (156).

As a novel echocardiographic technique, myocardial strain analysis can detect even subclinical myocardial dysfunction. Strain parameters are measures of myocardial deformation throughout the cardiac cycle. Strain analysis can be applied to various cardiac chambers to assess their function, including left atrium (LA) and LV. Global longitudinal strain (GLS) of LV and LA longitudinal strain are among the most studies and validated strain parameters.

**Figure 4:**
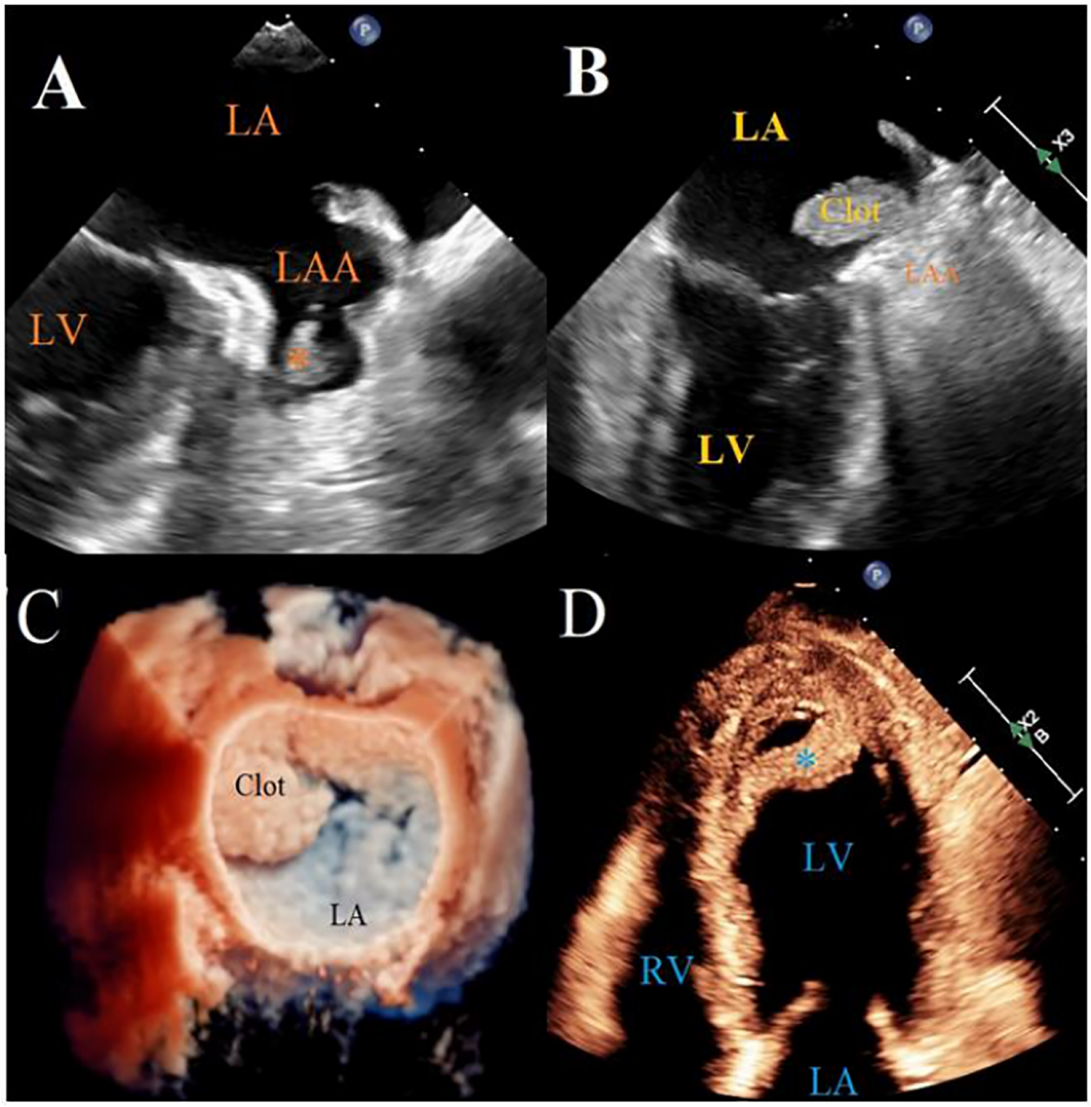
Left atrial appendage thrombus on TTE and TEE. **(A)** TEE image of a small LAA thrombus * in patient with AF. **(B,C)** 2D and 3D TEE reconstruction of a large thrombus protruding from LAA into the left atrium in a patient with factor V Leiden and AF. **(D)** TTE image of an apical LV thrombus *, detaching from the LV apex in a patient with acute myocardial infarction. TEE, transesophageal echocardiography; TTE, transthoracic echocardiography; LAA, left atrial appendage; LV, left ventricle; AF, atrial fibrillation.

LA strain analysis has been the center of attention lately. Abnormal LA strain parameters have been studied as potential independent risk factors for AIS, regardless of the presence of AF (156). This relationship is multifactorial and includes fibrotic remodeling of the left atrial walls leading to reduced output, and increased risk of thrombus formation, particularly in the left atrial appendage (Figure 4). This risk is particularly increased among patients with chronic atrial fibrillation and atrial flutter. While LV function can be assessed by LV strain analysis, left atrial strain is also associated with, and may be a proxy for LV dysfunction. In patients with reduced cardiac pump function, known as heart failure with reduced ejection fraction, (HFrEF), the hypokinesis of the ventricular walls leads to the risk of blood stasis, a key tenet of Virchow's triad, and further increases the risk of thrombus formation within LV cavity (157). AI/ML applications have been promising in timely detection of cardiac dysfunction, and therefore, allow for optimization of medical management to pre-empt the sequelae of thromboembolic events and AIS.

Therefore, access to AI-enabled tools for the early detection of cardiac dysfunction among high-risk patients and the timely diagnosis and management of HF may mitigate the risk of subsequent AIS. The recent advancement in AI applications has allowed for a growing role in the use of various algorithms for a myriad of cardiac conditions (158–160). Various AI/ML algorithms have been developed and implemented to utilize data from echocardiograms and EKGs for risk stratification, diagnosis, and prognostication for HF patients, particularly with respect to the heart-brain axis. As this technology continues to develop and be further incorporated into commercial use, it provides the opportunity to further revolutionize and increase the efficiency of detection and prognostication of HF. Moreover, its potential in detecting subtle cardiac abnormalities allows for the early screening and management of other cardiac conditions, which itself may prevent progression to HF and ultimately mitigate a risk factor of AIS.

#### 2.4.1 Diagnostic electrocardiography

The cardiac remodeling seen in HFpEF often manifests as EKG changes, a modality critical to the assessment of HF (161, 162). However, these subtle changes are often difficult to detect, which is why EKGs had not previously been an optimal screening mechanism for HF. Various studies have generated AI networks based on waveforms from EKGs to detect cardiac pathology ranging from ejection fraction to valvular disease, each implicated either directly or indirectly to HF (160, 163). The rationale is such that deep learning algorithms and neural networks could be “trained” with enough data to detect the otherwise subtle EKG abnormalities associated with the pathological cardiac remodeling seen in HF.

Akbilgic et al. developed an EKG-AI model using a deep residual CNN based on standard 12-lead EKGs (164). The authors noted that this model could predict HF with moderately high accuracy, with the authors noting an AUC comparable to AUCs from notable HF risk calculators from the Framingham Heart Study (FHS) and Atherosclerosis Risk in Communities (ARIC) study (164).

Other CNNs have been developed to detect HFrEF (with an EF of 40% or lower) with 12-lead EKGs data, with the capacity to “retrain” to utilize single-lead EKG input alone during routine examination, one of which is AI-EKG (165). Utilizing an EKG-enabled stethoscope, this deep learning system was able to detect HFrEF with an AUC of 0.81, a sensitivity of 91.9% and specificity of 80.2% (165). Kwon and colleagues also developed a deep learning model to predict HFpEF, based on an ensemble network using 12-lead, 6-lead, and single-lead EKGs. The authors noted a high level of predictive value (AUC 0.866) in detecting HFpEF using both 6-lead and 12-lead EKGs and noted that this model outperformed already-implemented screening-tests for other medical conditions, such as mammography for breast cancer (161).

##### 2.4.1.1 Vision transformer algorithms

Investigators have built on the above findings to generate vision-based transformer models such as HeartBEiT ® for EKG waveform analysis (166). Transformer based neural networks (TNNs) allow for the use of input (tokens) to develop relationships within and between data sizes (166). The development of Bidirectional Encoder representation from Image Transformers (BEiT) allows for the use of an input image to generate these tokens for input to be used to “train” the transformer, akin to words of a sentence for a language model. These tokens are used to generate downstream derivatives and output such as MI, left ventricular ejection fraction (LVEF), and hypertrophic cardiomyopathy. HeartBEiT ®, using these TNNs, has yielded a significantly higher performance in diagnosing the above conditions compared to standard CNNs, even with low sample sizes (166). The use of vision transformers presents a novel avenue of AI which warrants further investigation for its use in predicting and diagnosing HF.

#### 2.4.2 Diagnostic transthoracic echocardiography

In the HF care field, wearable sensors coupled with ML analytics may potentially improve clinical outcomes and reduce hospitalizations. One HF-specific, AI-powered platform which has recently gained approval from the FDA is the EchoGo® Heart Failure platform by Ultromics, based in the United Kingdom (UK). This platform applies AI via CNN to detect HFpEF using just a single 4-chamber TTE image. This apparatus was able to successfully reclassify HF among 73.5% of previously non-diagnostic results, with an accuracy of 90%, and a sensitivity and specificity of 87.8% and 83.0%, respectively (167). Moreover, Us2.ai, a Singapore-based medical technology firm has received FDA clearance for their automated decision support tool for echocardiography (168). LVEF is among the twenty-three validated parameters which can be used to assess cardiac systolic function. Such platforms allow for the accurate acquisition of cardiac measurements even without human input and may better optimize the screening and diagnosis of heart failure. There has also been an investigation done in the prediction of LAT using TEE vs. TTE images. Piezko et al. have done the most substantive investigation of AI in predicting LAT using TTE and clinical data compared to TEE prior to cardioversion or ablation (169). Their AI model accurately predicted LAT (AUC 0.85), outperforming other risk factors (LVEF and CHA2DS2-VASc), and would have avoided TEE in 40% of patients (169). While reducing TEE burdens, this also provides a means of significantly mitigating the risk of ischemic stroke in the future from said thrombi, particularly in an ever-growing cohort of patients with AF and atrial flutter.

With regard to myocardial strain analysis, motion estimation is the critical task to obtain GLS, which is a measure of LV function. This has conventionally been done using speckle tracking algorithms. Using echocardiograms, Salte and colleagues generated a deep learning AI pipeline consisting of 4 ANNs trained for motion estimation and compared this to semi-automated speckle-tracking (170). This pipeline was “taught” to detect patterns in consecutive images to obtain an optical flow vector field to predict local velocities, perform timing of cardiac events, and to visualize cardiac motion (170). Without any human input, this pipeline was able to successfully classify cardiac views, estimate motion, and ultimately measure GLS within 15 s, with accuracy comparable to conventional speckle-tracking methods (170). Therefore, the role of AI applications to assess cardiac function by performing myocardial strain analysis without human input, provides another means of expedited, more efficient detection of HF, and warrants further investigation.

### 2.5 Cardiac tumors

While cardiac tumors are a comparatively less common form of cardiac pathology, they still confer significant morbidity and mortality if not detected. Within neurocardiology, this is especially relevant in the setting of cardiac myxomas and papillary fibroelastomas which can embolize from the left atrium or the valvular endocardium and cause large-vessel occlusions (LVOs) in the brain (171). Various imaging modalities are used to evaluate and diagnose cardiac tumors (171). These include TTE, TEE, cardiac MRI, cardiac CT, and positron-emission tomography (PET) scans (172). The imaging modality of choice depends greatly on tumor characteristics such as whether the tumors are primary vs. metastatic lesions and of tumor location (right heart vs. left heart) (172). To this point, the current literature surrounding the role of AI and ML for diagnosing cardiac tumors is relatively limited. To date, there are no studies which have demonstrated efficacy for the utilization of AI in cardiac tumor evaluation.

#### 2.5.1 Diagnostic parallel computing

One avenue of potential inquiry in the context of detecting cardiac tumors using AI is that of parallel computing to identify cardiac tumors. Parallel computing entails the use of computers to perform multiple tasks simultaneously to break a multi-faceted problem into individual tasks to be solved (173, 174). In the context of cardiac tumors, this would involve segmenting the various radiographic features of the tumor across multiple imaging modalities and incorporating this data to generate a single diagnosis (173). Computing may be supplemented and enhanced by AI and ML algorithms to improve the sensitivity and specificity of current imaging modalities for diagnosing cardiac tumors (174, 175). In the realm of neurocardiology, this is particularly pertinent with respect to myxomas which may embolize and lead to LVOs and infarcts (176).

#### 2.5.2 Stroke prediction

AI models have increasingly been used to predict the risk of stroke amongst patients with specific comorbidities (177, 178). Despite the elevated risk of stroke with cardiac tumors such as myxomas and papillary fibroelastomas, there are currently no studies evaluating the risk of stroke in patients with any form of cardiac tumors. This represents an important gap in the literature and should be an area of focus in future studies evaluating the role of AI in stroke prediction.

### 2.6 Infective endocarditis

Infective endocarditis (IE) is a sequela of systemic bacterial infection spreading to the endocardium, the inner lining of cardiac muscle and valves. IE can have severe systemic effects on a myriad of organ systems, with the nervous system being the second most affected (179). The neurological manifestations of IE from embolism of valvular vegetations include embolic stroke, cerebral hemorrhage often in the setting of mycotic aneurysms, and the spread of infection leading to meningitis, brain abscesses, and toxic encephalopathy (172, 180).

Among the most common causes of morbidity and mortality in the setting of IE is ischemic stroke. Strokes secondary to cerebral emboli represent up to 42% of neurologic complications in IE patients (181). Vegetations on cardiac valves have a propensity to embolize into large cerebral arteries and disrupt perfusion to various parts of the brain (182). Therefore, timely diagnosis and treatment of IE can reduce the risk of embolization to the brain and LVOs which would otherwise require mechanical thrombectomy. According to the literature, there is a threefold decrease in stroke risk within the first week of treating IE with antibiotics (182, 183). Therefore, early detection and treatment of IE even before overt systemic manifestations is essential, with at least 30% of IE patients developing subclinical neurological manifestations such as silent cerebral emboli (184).

#### 2.6.1 Diagnostic 18f-fluorodeoxyglucose PET/CT

18F-Fluorodeoxyglucose (FDG) PET/CT involves injecting a radiolabeled glucose analog into the patient and evaluating its uptake throughout the endocardium, myocardium, and other cardiac structures. Radiotracer uptake is higher in metabolically active tissues, as in the case of IE where FDG PET/CT can visualize infected valvular structures with high uptake (185). This technique is most useful to rule out IE in prosthetic heart valves, where other imaging modalities including echocardiography may be extremely limited. However, the lack of standardized metrics of uptake and the difficult distinction between infection and inflammation has limited its use (185, 186). The integration of AI enabled better differentiation among various cardiac pathologies such as valvular abscesses, intracardiac fistulae, and pseudoaneurysms seen in IE, as opposed to reactive inflammatory changes seen either postoperatively or in the setting of chronic valvular disease.

In 2016, Godefroy et al. assessed AI and ML algorithms with FDG PET/CT in the setting of IE (187). Using a sample of 68 patients with a known prior history of IE, the authors developed an AI and ML algorithm model that could be applied to 40 patients with suspected IE in their study (187). The model was combined with the European Society of Cardiology's 2015 criteria for suspected IE. The AI model demonstrated higher rates of accuracy, sensitivity, and specificity when compared to expert clinicians alone (183). Unfortunately, few studies analyzing AI and ML for imaging in IE have been published since, which has stymied the advancement of AI and ML as diagnostic tools for IE patients.

#### 2.6.2 Stroke prediction

Resende et al. developed a log-linear ML model which was able to predict higher rates of inpatient mortality amongst IE patients undergoing surgery compared to patients who received conservative management (188). Another study by Luo et al. had similar results with a ML model that predicted early mortality and risk of stroke after surgery better than pre-existing logistic regression models and the well-established European System for Cardiac Operative Risk Evaluation model (189).

However, while there have been very few investigations focusing specifically on AI/ML applications for neurological sequelae, the prospective use of AI/ML serves as “proof of principle” that prevention is better than cure. Neurological conditions are among the most common manifestations of systemic IE, and therefore, the focus of using AI/ML technology in the realm of neurocardiology for IE patients is best suited to detection of infective cardiac pathology before the neurological pathologies manifest.

## 3 AI/ML applications in strokes of undetermined etiology

### 3.1 Cryptogenic stroke

Cryptogenic stroke (CS)/ESUS remains a significant clinical challenge not only due to its complex and multifactorial etiology, but also due to its burden of comprising 20%–30% of all ischemic strokes in the TOAST classification. While technically termed due to its inability to locate a clear etiology, possible sources of cryptogenic stroke include pathologies such as paroxysmal atrial fibrillation, paradoxical emboli through PFO, non-stenotic carotid plaques and atrial cardiopathy (37, 38, 77, 190). Many studies report these diagnoses can be found with comprehensive or extended cardiac monitoring instead of standard evaluation protocols (21, 28–38) For example, AF may comprise up to 15% of all cryptogenic strokes and the prevalence of PFO is reportedly as high as 57% (26, 32, 79, 147).

Currently, cryptogenic stroke is largely a diagnosis of exclusion during comprehensive neurocardiology workup. These include neuroimaging studies such as MRI or CT, cardiac monitoring in the form of standard 12-lead EKG, Holter monitoring or extended cardiac monitoring such as implantable loop recorders to detect occult AF, TTE, TEE to assess for embolic sources such as intracardiac thrombi or PFOs, and blood tests to determine coagulability status and the presence and severity other pre-existing metabolic disorders (37, 38, 190, 191).

AI provides another avenue of investigation: reducing repeat stroke events and future morbidity for stroke patients. For example RESPECT and CLOSE trials have provided evidence supporting the closure of PFO in selected cryptogenic stroke patients and demonstrated significant reductions in recurrent stroke rates compared to medical therapy alone (192, 193). These trials underscore the importance of individualized treatment approaches based on specific etiologic factors identified during diagnostic evaluations.

#### 3.1.1 Diagnostic electrocardiography

A four-year retrospective study conducted with patients diagnosed with cryptogenic stroke from 4 Korean tertiary hospitals found that an image-based AI model of EKG data had excellent diagnostic performance in AF prediction. The AI-AF risk score, derived from sinus rhythm EKGs, demonstrated high predictive accuracy for AF, with an AUC of 0.806. Incorporation of parameters such as left atrial volume index, age, sex, BMI, atrial ectopic burden, and PR interval further improved the model's performance (AUC 0.880, *p* = 0.002). There was also a significant temporal trend showing increased AI-AF score with increased AF duration and as recorded EKG time approached AF onset (37). This AI-based approach not only aids in early AF detection, but also offers potential timesaving and cost-effective advantages for timely interventions to prevent recurrent strokes.

These platforms can utilize EKG data to retrospectively detect undiagnosed AF in cryptogenic stroke patients. Even for patients who already have suffered from cryptogenic stroke, AI can detect AF as an etiology for cryptogenic stroke, thereby allowing for secondary prevention of future ischemic strokes. Choi et al. recently developed a transformer-based AI model trained to analyze EKGs from cryptogenic stroke patients both to detect and predict paroxysmal AF (37). This model demonstrated a robust performance, with an AUC of 0.880, and a greater accuracy in identifying longer duration AF episodes (37). Ho et al.'s meta-analysis found multiple new wearable technology, that includes those currently being used after cardioembolic stroke, to be feasible options for diagnosis of paroxysmal atrial fibrillation in cryptogenic stroke (194). However, they noted no current significant difference of these modalities compared to classic workup technology (such as Holter monitoring) and emphasized the need for further studies.

Comparing the efficacy of wearable technology for cardiac monitoring after stroke found there were promising technologies in EKG and PPG data as previously described for afib.

#### 3.1.2 Diagnostic language processing

Investigators such as Garg et al. have demonstrated that ML models with natural language processing (NLP) may provide an avenue of automation for the classification of ischemic stroke subtypes based on data from patient electronic medical records (EMR), medical history, physical examinations, laboratory and imaging results (77). These authors tested various ML models such as K-nearest neighbors (KNN), random forests (RF), and XGBoost and conducted a stacking process, whereby model predictions are used to generate new models. They found an 80% agreement between a stroke neurologist and the ML model using stacking for logistic regression with kappa of 0.72 for TOAST classification, with excellent subtype discrimination in all ML models (c-statistic >0.8) aside from the KNN approach. This study suggests that ML models can not only enhance scalability of large-scale epidemiological studies and clinical trials, but also remove a point of error in manual adjudication of disease classification and investigator bias (77). For cryptogenic stroke, ML models may help in analyzing etiologic phenotypes leading to further subtyping for directed treatment.

Using AI tools such as the NLP machine learning model to automate subtype classification in ischemic stroke can significantly impact management strategy based on characteristics found in various clinical studies (77). Management of cryptogenic stroke currently focuses on secondary prevention to reduce the risk of recurrence. Risk stratification tools, including the AI-based models listed above, can guide physician decisions on anticoagulation vs. antiplatelet therapy for patients with or without identified sources of cardioembolism, such as AF or a PFO (190).

Additionally, the CHA_2_DS_2_-VASc score, traditionally used to predict stroke severity in patients with AF, is now being considered in patients with cryptogenic stroke and underlying congenital heart disease (CHD) (144).

#### 3.1.3 Diagnostic imaging

Detection of congenital heart abnormalities in cryptogenic strokes include the use of modalities such as TTE, TEE, cardiac MRI, cardiac CT, and transcranial doppler ultrasound (TCD). TEE with agitated saline injection remains the gold standard for PFO diagnosis relative to intraoperative inspection, with a weighted sensitivity of 89% (149). The presence of PFO in cryptogenic stroke patients has been associated with various morphologic and functional characteristics, including the size of the PFO, mobility of the interatrial septum, and the presence of a right-to-left cardiac shunt.

The CHALLENGE ESUS/CS registry, a multicenter registry of cryptogenic stroke patients, utilized TEE to identify potential embolic sources (147). While there are advantages to cardiac MRI for detecting certain cardioembolic sources, a meta-analysis showed a 29.3% (95% CI, 23.6%–35.0%) detection yield for CMRI compared to TEE at 53.7% (95% CI, 47.4%–49.9%) (*P* < 0.001) (149).

For PFOs, Bai et al. found that, in cryptogenic stroke patients, PFO size was significantly greater in systole [2.0 (1.5, 2.9) mm vs. 1.6 (1.1, 2.0) mm, *p* < 0.001] and diastole [1.7 (1.4, 2.2) mm vs. 1.3 (1.1, 1.8) mm, *p* < 0.001] than in non-CS patients and that large PFO were also more common (*p* < 0.001) (146). ANN and SVM were performed as comparisons with goodness of fit testing and coefficient of certainty, with higher accuracy and reliability noted with the RF model. The final test set accuracy was 70%, with an AUC of 0.816 and a sensitivity and specificity of 73% and 65%, respectively. Additionally, the frequency of right-to-left shunt was significantly higher in CS patients (*p* < 0.001) (146). Supervised RF learning is insensitive to noise with good generalizability, making it suitable for the high-risk features of PFO.

### 3.2 Atrial cardiopathy

Atrial cardiopathy represents pathological structural changes of the atria due to stretching from progressive cardiac stress. This occurs in response to various stressors such as hypertension, heart failure, obesity, valvular heart disease, etc. Atrial remodeling then leads to degenerative changes such as atrial dilation, fibrosis, and myocyte damage (195). The pathophysiology of atrial cardiopathy both precedes and is related to AF and its medical sequela (195). Specific to neurocardiology, left atrial remodeling can induce thrombus formation and subsequent embolization and strokes, even in the absence of dysrhythmias such as AF (195). In fact, AF itself can manifest secondary to this remodeling and perpetuate the risk of cardioembolic strokes. Therefore, AF can be viewed as one element of a larger overarching pathological cardiac disease process of atrial cardiopathy (196). EKG analyses have shown up to50% of stroke patients demonstrate atrial abnormalities on EKG even without AF (197). Therefore, a more holistic evaluation of atrial pathology, ranging from EKG findings such as P terminal force in lead V1 (PTFV1), to echocardiographic parameters such as atrial size, and biomarkers such as NT-proBNP may allow for a more thorough assessment of embolic stroke risk than AF alone (196).

#### 3.2.1 Diagnostic electrocardiography

AI may facilitate the use of ML to analyze diagnostic modalities for the early detection of atrial pathology, thereby mitigating the risk of AF and of subsequent cardioembolic and cryptogenic strokes. Additionally, it can allow for detection of undiagnosed AF to reduce the risk for future cardioembolic events even for patients who already suffered from strokes.

Studies into AI for atrial cardiomyopathy have recently been conducted with the rationale that early progression of the disease process can manifest as subtle EKG changes which can be detected via ML. Investigators at the Mayo Clinic developed an AI algorithm consisting of a CNN trained to screen for temporal and spatial features on 12-lead EKGs to identify and stratify atrial cardiomyopathy for patients with heart failure with preserved ejection fraction (HFpEF) (198). This algorithm was able to identify structural heart disease in patients with higher AI-probability for AF based on EKG findings, with these patients demonstrating LVH, increased atrial filling pressures, and reduced cardiac output (198).

Therefore, while AI is still in its relative infancy for use in atrial cardiopathy, these algorithms provide a means of primary, secondary, and tertiary prevention. The timely detection of subtle cardiac structural and hemodynamic changes can allow for clinicians to intervene before the manifestation of further cardiac pathologies such as AF and subsequent stroke.

## 4 The brain-heart-axis: AI/ML applications in cardiac disease secondary to stroke

### 4.1 Neurogenic stress cardiomyopathy (takotsubo syndrome)

Neurogenic Stress Cardiomyopathy (NSC) is the manifestation of catecholamine-mediated myocardial dysfunction in the setting of acute brain injury, ranging from subarachnoid hemorrhage (SAH) to stroke (199, 200). CNS pathology secondary to stroke is posited to induce perturbations to the hypothalamic-pituitary-adrenal (HPA) axis, leading to sympathetic activation and downstream local and systemic inflammatory cascades (199, 200). This leads to coronary microvascular spasm, oxidative stress, myocardial inflammation, and myocyte injury. This manifests diagnostically through EKG changes such as ST segment changes, and QT prolongation, distinct patterns of LV wall motion abnormalities and elevation in cardiac troponin and NT-pro brain natriuretic peptide (BNP) levels (199, 200).

**Figure 5:**
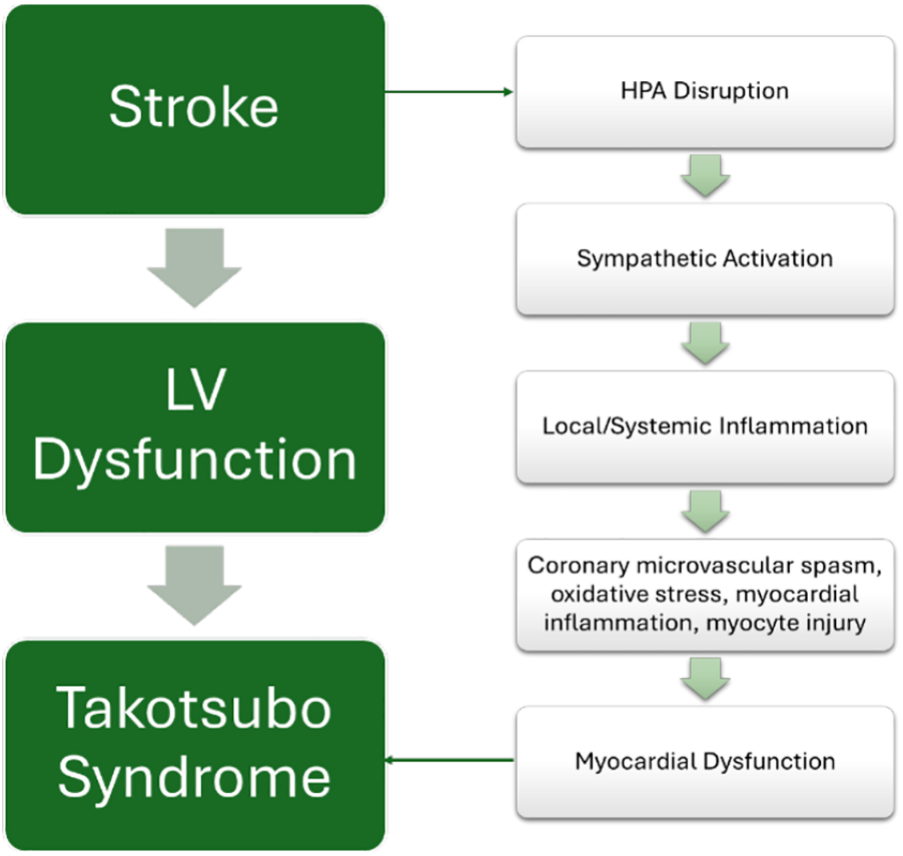
Utilizing neurocardiology to understand the pathophysiology of Takotsubo syndrome.

**Figure 6:**
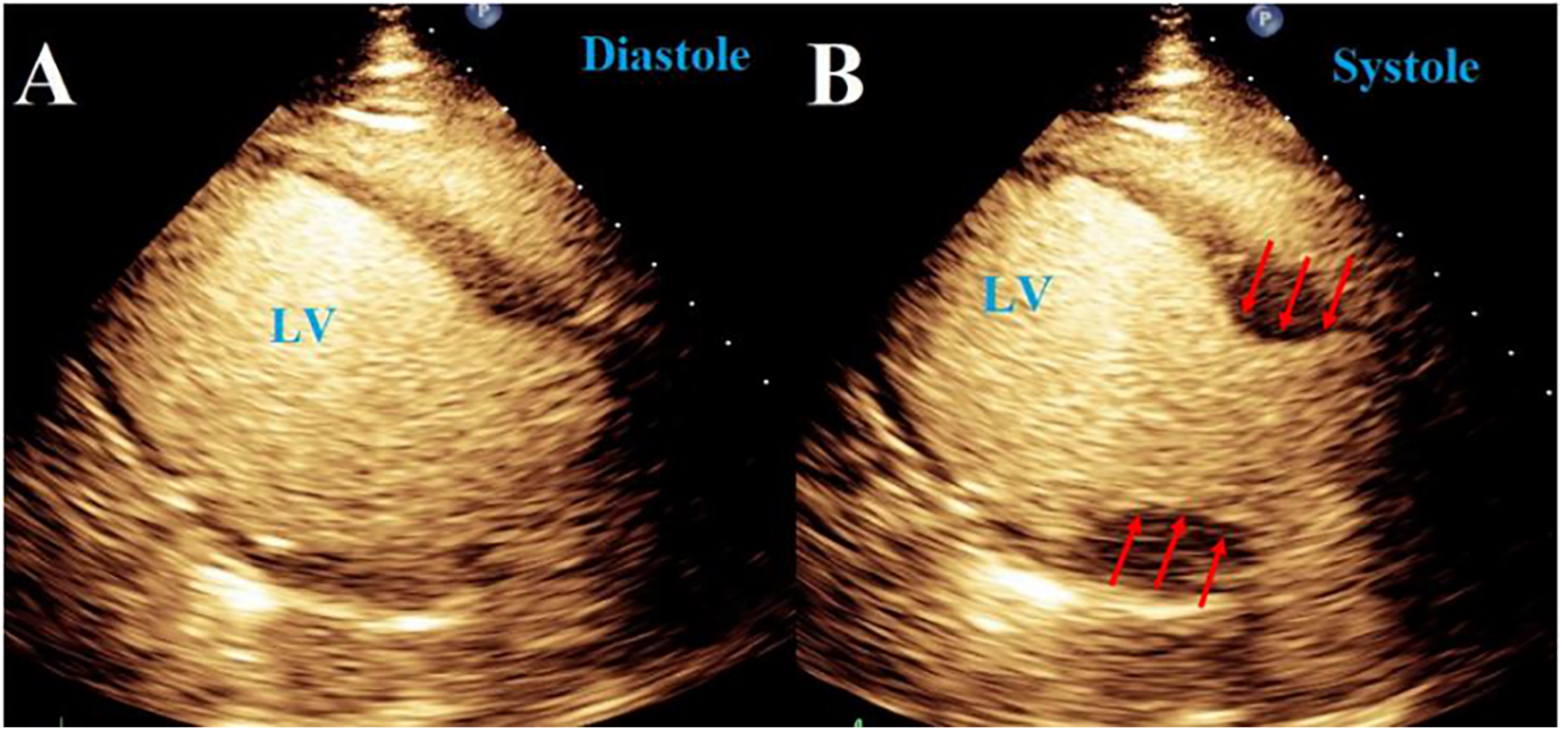
Takotsubo syndrome. **(A,B)** 2D TTE images with echo-contrast agent of “Takotsubo cardiomyopathy” demonstrating a distinct pattern of left ventricular wall motion abnormality known as “apical ballooning” in a patient with Subarachnoid hemorrhage (SAH).

NSC is proof of concept of the heart-brain axis (Figure 5), with authors referring to NSC as “Takotsubo syndrome secondary to neurological disorders” (201). Takotsubo cardiomyopathy (TTC) is recognized as a reversible LV dysfunction in the setting of stress, named for the apical ballooning of the heart on ventriculography which resembles a Japanese octopus trap pot (Figure 6) (202). A common cardiac complication for patients with neurological disorders in the intensive care unit (ICU), TTC closely mimics the clinical presentation of acute coronary syndrome (ACS), with ST-segment elevations on EKG with or without elevated troponins, making initial diagnosis difficult. It is ultimately differentiated from MI by excluding occlusive coronary artery disease (201, 202).

The role and duration of conventional guideline-directed HF medical therapy is controversial in TTC. TTC often responds to supportive medical management, however, occasionally may lead to cardiogenic shock requiring hemodynamic support. Overall, with timely and efficient medical management, outcomes from TTC are generally favorable and the systolic dysfunction is often reversible (203). Rare complications of TTC including fatal LV rupture and LV thrombi have been reported, the latter cited in 5% of TTC patients (204). LV thrombi in turn, can result in further cardioembolic strokes. According to the literature, TTC complicated by LV thrombi, is associated with very high risk of stroke of up to 23% (205).

#### 4.1.1 Diagnostic transthoracic echocardiogram

Echocardiography is the primary imaging modality, used to diagnose TTC. However, initial differentiation between ACS/MI and TTC remains challenging. Therefore, investigators have sought to develop algorithms to utilize echocardiograms to differentiate TTC from MI. Laumer and colleagues developed and trained a DL algorithm using TTEs from 228 patients to differentiate TTC from acute MI (206). They utilized a temporal CNN to predict the probabilities of TTC and MI for the respective patients and compared these findings to echocardiographic evaluation by 4 cardiologists (206). This algorithm demonstrated a superior performance to the committee of cardiologists in differentiating TTC from acute MI, with an AUC of 0.79 and an overall accuracy of 74.8% (compared to an AUC 0.71 and an accuracy of 64.4% for the cardiologists) (206).

#### 4.1.2 Diagnostic cardiac MRI

While TTE has traditionally been a first-line imaging modality for TTC, cardiac MRI is emerging as a gold standard, non-invasive modality to differentiate TTC from acute MI. For instance, T2-weighted imaging can detect myocardial edema, which manifests in the acute phase of TTC and is indicative of reversible myocardial injury (207). Other MRI criteria for TTC include wall motion abnormalities and the relative lack of contrast enhancement (208). Additionally, atrial and ventricular strain parameters are of growing relevance to diagnosing TTC, and MRI provides an ideal modality to conduct strain analyses. Authors have also utilized ML with cardiac MRI protocols to diagnose and differentiate TTC from other cardiac pathologies. Cau et al. created a cardiac MRI-based ML ensemble model using the Extremely Randomized Trees algorithm trained with MRI strain measurements and demographics to different TTC from myocarditis. The authors noted a high degree of sensitivity (92%) and specificity (86%) with an AUC of 0.94 in diagnosing TTC (209). The authors also noted that strain rate and left atrial conduit strain were the parameters that yielded the greatest value for TTC identification (209).

#### 4.1.3 Stroke prediction

Investigators have developed AI algorithms for stroke risk prediction in TTC. De Filippo and colleagues analyzed patients from the International Takotsubo (InterTAK) registry in a multi-center study and designed their own ML model to predict in-hospital mortality (210). This was a logistic regression-based algorithm evaluated in internal and external validation cohorts (210). This InterTAK model demonstrated a strong performance in predicting mortality for the internal validation cohort, with an AUC of 0.89, a sensitivity of 85% and a specificity of 76% in the internal validation cohort (210). The model also yielded satisfactory performance on the external validation cohort, with an AUC of 0.82, a sensitivity of 74%, and a specificity of 79% (210). The authors also showed the further applicability of ML: AI for subsequent regression analyses to ascertain patient characteristics also associated with mortality. They noted that LVEF, atrial fibrillation, physical stress, leukocytosis, and age were among the most relevant features associated with mortality. From these ML-based analyses, the authors were able to cluster patients into six risk groups based on short-term prognoses (210).

##### 4.1.3.1 Cardiac MRI

In line with the growing use of cardiac MRI for diagnosis of TTC, researchers have proposed the applicability of MRI with AI to predict major adverse cardiac and cerebrovascular events (MACCEs) in TTC patients (211). This potential application is part of a larger field of radiomics, in which algorithms are generated to extract quantitative metrics from imaging. Within this field, texture analysis (TA) can be used to evaluate the texture of images to diagnose imaging abnormalities which otherwise could not be seen by the naked eye (211). ML provides a platform to scope through large volumes of data to detect imaging patterns for prognostication. Mannill and colleagues applied this concept using MRIs with cine sequences providing visualization of cardiac wall abnormalities for 58 InterTAK registry patients (278). From these images, they assessed 5-year outcomes for MACCEs using various machine-learning classifiers ranging from ANN Multilayer Percepton, to NaïveBayes (211). NaïveBayes demonstrated the most robust performance, with an AUC of 0.88, sensitivity of 82.9%, and a specificity of 83.7% (211).

While these studies face the inherent caveats of small sample sizes and being retrospective in nature, they do provide proof-of-concept of the applicability of AI for TTC, both as an effective diagnostic and prognostic tool, avenues which requires further investigation. Being able to obtain prompt diagnoses and risk stratification for TTC can minimize the risk of future ischemic stroke events and subsequent cardiac pathology secondary to the perturbation of the bidirectional heart-brain axis.

### 4.2 Post-stroke arrythmias

While AF is one of the most common causes of AIS, AF also reflects the reciprocal relationship of the heart-brain axis as AF may itself develop secondary to AIS. Since AF commonly presents following a prior stroke, the question is whether this trend reflected previously undiagnosed AF (cardiogenic AF) or manifested due to new-onset myocardial injury as a sequela of cerebrovascular insult (neurogenic AF) (7, 9, 75, 212). Newly diagnosed atrial fibrillation (NDAF) after AIS has been reported to be found in 7%–25% of poststroke patients and is thought to be secondary to both autonomic dysregulation and inflammation resulting in reentry circuits and atrial remodeling (213–217). This is an additional area in which improved methods of diagnosis and monitoring, particularly by using AI to more accurately predict high risk patients, will likely increase rates of detection and incidence (218, 219).

#### 4.2.1 Diagnostic clinical analysis

There have been few validated scores developed to predict NDAF with a high degree of external validity, two of which include the CHASE-LESS and AS5F scores (220–224). However, various ML algorithms are currently being developed to provide a higher accuracy prognostic modality in this role (225–228). Sung et al. designed an ML algorithm to utilize stroke hospital admission data in the EMR to predict newly diagnosed AF early in patients’ hospital courses (227). This model utilized common EMR data fields and clinical free text information. It outperformed standard prediction methods (as shown by a higher C-index) (227). This type of automated risk predictor allows clinicians to treat cerebral ischemia while understanding the full clinical picture of the patient, encompassing the broader heart-brain axis.

AI models have also been generated using other markers, such as BNP, neuroimaging, and genetics. A 2022 study by Pang et al. developed and evaluated an integrated nomogram model for post-stroke AF based on variables such as prior TIA, BNP levels, CRP levels, and National Institute of Health Stroke (NIHSS) scores. This integrated model provided enhanced benefit predicting the incidence of post-stroke AF, identifying higher-risk patients who were candidates for extended monitoring (217).

### 4.3 Coronary artery disease

Coronary artery disease (CAD) is among the leading causes of death and disability worldwide, accounting for up to 31% of global mortality from non-communicable disease (229).

Given the inextricable link between cardiovascular and cerebrovascular disease in the heart-brain axis, ischemic stroke patients and patients with intracranial atherosclerotic disease (ICAD) are invariably at increased risk for concordant CAD and its potentially fatal manifestations such as MI (MI) (230). The Prevalence of Asymptomatic Coronary Artery Disease in Ischemic Stroke Patients (PRECORIS) study noted that almost a fifth of patients with ischemic stroke or TIA also had at least 50% obstructive coronary artery disease, even if asymptomatic (231). Therefore, the detection and prediction of silent CAD in stroke patients can facilitate timely medical management to prevent the progression to severe coronary artery disease and ACS.

Acute MI represents the most severe manifestation of CAD. The diagnostic protocol for suspected acute MI includes a standard 12-lead EKG, and while ST-elevation MI (STEMIs) can often be diagnosed quickly, non-ST elevation MIs (NSTEMIs) can demonstrate subtle changes on EKG in up to 30% of patients that may be missed even by the experts (232). Currently even the application of new generation high-sensitivity cardiac troponin assays, it might still take between 1 and 3 h to demonstrate a diagnostic rise or fall pattern on troponin levels which is necessary for diagnosis of acute MI (232). Therefore, there has been a growing enthusiasm in investigating the role of AI in EKG analyses as an adjunct to biomarkers for the timely diagnosis of AMI.

#### 4.3.1 Diagnostic electrocardiogram

Cho et al. developed a DL algorithm to use 6-lead EKGs to diagnose MIs, with reasonable accuracy (sensitivity 84.4%, specificity 88.5%) and high negative predictive value (97.5%) (233). This has undergone iterative development to extend to 12-lead EKGs. One Taiwanese retrospective study investigated a DL model trained detect STEMI through analysis of 100,000 EKGs and compared its performance to cardiologists. The authors noted a superior performance of the DL algorithm, with a high AUC of 0.997, sensitivity of 98.4%, and specificity of 96.9% (234).

Moreover, the Rule-Out Acute Myocardial Infarction Using Artificial Intelligence Electrocardiogram Analysis (ROMAIE) trial is among the major prospective clinical trials currently underway to validate a DL-based 12-lead EKG algorithm (235). This study will allow for real-time substantiation and further application of AI in a clinical setting for AMI.

There are relatively few studies to date which have analyzed AI algorithms in the detection and prognostication of CAD specifically in stroke patients. One such study was conducted by Heo et al., who obtained multidetector coronary CTAs for patients who suffered acute ischemic strokes without a known history of CAD (236). The authors obtained demographics, laboratory results, NIHSS scores, blood pressure, and carotid stenosis to generate an ML model to predict CAD (236). They noted a satisfactory performance in predicting CAD, with an AUC of 0.763 in predicting CAD of any degree, and an AUC of 0.714 to predict obstructive CAD (236). Other investigators have developed models to incorporate both DL and ML algorithms using a combination of EKG, clinical, and laboratory data to predict obstructive CAD, with similar results (AUC of 0.767) (237).

However, the results of AI investigations for broader patient cohorts have demonstrated the potential of AI as a diagnostic and prognostic tool for CAD. Kang et al. developed an SVM-based learning algorithm to utilize coronary CTAs to diagnose coronary artery stenosis and compared this algorithm to three expert readers of CTA imaging (238). This model showed a superior performance to the human readers with a high degree of accuracy (AUC 0.94, sensitivity of 93%, sensitivity of 95%) (238). While this model did not specifically focus on stroke patients, it demonstrates the potential of such algorithms as tools for diagnosis and prognostication of CAD which can be applied specifically for ischemic stroke patients in future studies.

## 5 Limitations and future directions: AI/ML applications in neurocardiology

### 5.1 Practical limitations

AI/ML provides a highly beneficial tool for disease prediction, identification, and prognosis through utilization of multiple data modalities for augmentation and personalization of clinical decision making. In neurocardiology, these tools are essential for identification of pathology between the nervous and cardiovascular systems and may facilitate better optimization and more timely management of modifiable comorbidities. AI/ML can improve speed of care and personalized assessment while also reducing human error and physician burnout (75, 80, 82–84).

However, with these potential benefits, the AL/ML tools reviewed here have several inherent limitations in the creation of their respective algorithms. Firstly, physician input is always required to ensure accuracy and to check algorithm training. This is often a tedious process and is a rate-limiting step in the progression of this technology. Secondly, most of these programs are designed with one specific outcome variable, such as the presence of atrial fibrillation or the risk of stroke recurrence. There are very few options currently available that can analyze multiple modalities of diagnostic imaging and generate diagnoses and prognoses for a broader differential of neurological and cardiovascular conditions. Therefore, these tools are still in their relative infancy and are no substitute for sound human clinical judgment.

Furthermore, physicians also encounter patient populations with varying degrees of digital literacy and access to technology which can directly impact health equity (239, 240). Sieck et al. have recently deemed digital inclusion to be a social determinant of health (241). As reliance on technology increases, so does the potential for disparities in access to care using this technology. This provides practical limitations of AI/ML options that involve patient interaction and challenges providers to be able to engage in community programs or provide equivalent patient care through different modalities (241–243).

While AI can serve to bridge this gap through user friendly technology and personalized assistance with ML, these tools also possess the potential for patient bias that may disadvantage certain populations (244). This problem can often be caused by the limited generalizability of single center studies along with observed racial biases in algorithms due to different healthcare needs and trends for various patient populations (244, 245, 274).

Another significant limitation in the application of AI/ML in healthcare is physician willingness both to be educated in its use and to incorporate it into broader clinical practice. Many studies have looked at physician and technician perspectives on the integration of this technology. While most studies cite at least 70% approval from the physician community, there remains a sizable contingent who are more reticent to incorporate AI into their practice. This hesitancy is multifactorial, ranging from ethical concerns to insufficient understanding of how to use the technology (246–249). However, the greatest concern among physicians is the fear of overreliance on AI which may be to the detriment of clinical judgment, as well as medical error liability secondary to errors in automation (246–248, 250, 251).

Health care providers and students would therefore require proper training in using AI/ML. While the rigor of this training may vary depending on the AI application, there is significant support for formal training programs at all education levels (247, 252–255). Karaca et al. have suggested the use of a psychometric measurement tool called the Medical Artificial Intelligence Readiness Scale for Medical Students (MAIRS-MS) to guide assessment in education on AI (256). There has also been research regarding the creation of ethics and public health-based AI education for students and providers (257). The literature notes that while AI has many benefits, those are limited by the ability to standardize its implementation and train clinicians to effectively use it (82).

### 5.2 Ethical implications

The ethical implications of AI/ML use in healthcare raises several questions, such as the limitations of its use mentioned above, privacy/confidentiality of data, creation of federal standards and guidelines, and both physician and patient transparency in the process (258, 259). Preserving the confidentiality of data is an ongoing challenge since the use of electronic health records, with concerns such as hacking and data breaches. However, some risks unique to AI have been mitigated with local generative models and federated learning models (258, 260, 261).

The first set of international guidelines for clinical trials using AI was developed in 2021 with the SPIRIT-AI and CONSORT-AI guidelines to help standardize development and reporting methods of new algorithms for disease detection and diagnosis (258, 262). Most recently in 2024, the World Health Investigations (WHO) released their recommendations on suggested governance for AI models in medicine (263).

The “Black Box problem” with AI/ML describes the lack of understanding in the general population for the actual decision-making process that occurs in the algorithm (258, 264, 265). One solution to this is the creation of “explainable AI” that provides more user-friendly algorithms with explanations for the decision (265–270). While these may provide a better integration of human interaction within the algorithm, it also means it is more labor intensive and may not provide the same efficiency as AI nor the rapid growth and adaptability of ML (270).

Xu et al. note the underestimation of the possible malpractice and harm that could come to patients from AI in the forms of lack of proper informed consent and increased psychological and financial burden of these systems (265). The authors cite the need for physicians to understand where and how AI should be implemented, its limitations, and the continued development of quality control measures to ensure standardization (265).

While these ethical concerns are important to both health care workers and patients, it is also important to recognize the clinical value of AI/ML which has been iteratively integrated into clinical practice over the last several decades. The advancement of this technology has continued to drive the evolution of modern medicine. With proper regulatory oversight, patient-focused approaches, and training to understand the benefits and limitations of AI, this technology may potentially revolutionize how we assess and manage patients in both neurocardiology and medicine overall.

### 5.3 Future directions

#### 5.3.1 Future clinical applications

There have already been substantive investigations conducted in assessing the use of AI/ML, DL, or CNN algorithms for the timely detection and prognostication of acute ischemic stroke, cardiovascular diseases, and neurological pathologies. These studies have demonstrated an iterative increase in accuracy, speed, and applicability of this technology within diverse settings. The recent algorithms discussed have also proven their strong statistical power in evaluating complex datasets and identifying trends that may otherwise elude traditional statistical analysis.

These advancements in clinical use originate from the ability to incorporate anatomic analysis, functional and hemodynamic parameters, clinical factors, and imaging modalities. Mobile devices and other widely sold technologies continue to provide greater ease of monitoring, more timely diagnosis, and therefore, more timely treatment. Simply having increased accessibility to continuous monitoring may also increase the predictive value of them. These uses thereby may improve patient outcomes and reduce the overall burden on the healthcare system.

As well as its application in standard clinical decision making, future directions using AI/ML include improving access to more equitable healthcare to underserved communities (82, 85, 86). This may be accomplished through increased digital access to diagnostic tools, improving efficient clinical decision making for disease escalation, and reducing physical or cultural barriers through access to high quality care with telemedicine (86, 244, 271). Some of these goals are already evident with common technology today, through systems such as broadened Medicare coverage for telestroke (272). Technology can also provide greater autonomy to patients with managing their own health needs, such as the tools above which allow for cardiac monitoring even at home or in clinic (273). Furthermore, substantive research has been conducted to address the limitations of bias evident in AI algorithms with suggestions of improvement including careful cohort selection, cultural awareness and expertise in developing algorithms, and joint multicenter data (271, 274–277).

There is a clear demand for expanded clinical use of these AI-enabled platforms and algorithms. Robust validation of these ML/AI models is required for future clinical use, as well as ensuring data privacy and security, addressing algorithm bias and interpretability, and future studies on multi-center populations. The widespread incorporation of these models may also offer an avenue to accelerate the development of better diagnostic and predictive technology.

#### 5.3.2 Future research directions

There is an evident difference in the depth of AI/ML research conducted between certain neuroradiology pathologies. Of note, the investigation of this technology remains limited for cardiac tumors, the detection and prognostication of CAD in stroke patients, and in the neurological sequelae of infectious endocarditis, such as meningitis, abscesses, mycotic aneurysms, and intracerebral hemorrhage. Further studies are warranted to expand on the prospective benefits of AI/ML applications in these and other related cardiovascular and cerebrovascular conditions.

The future of AI/ML in neurocardiology is promising, particularly in the development of future investigations that may provide further diagnostic and prognostic value for stroke patients, better elucidate the sequelae of presenting and developing diseases, and improve patient care with its increased efficacy. As AI/ML becomes more commonly incorporated into clinical practice, the knowledge base, algorithmic training, and accuracy of models will continue to improve.

## 6 Conclusion

The heart-brain and brain-heart axes encompass the many clinical features and interconnected pathophysiology between cardiac and cerebrovascular diseases. Assessment and management of stroke patients should take place within the larger framework of neurocardiology, through initial stroke prevention for patients with cardiac disease, cardiac evaluation for stroke patients, and secondary stroke prevention for patients with newly diagnosed, post-stroke cardiac disease. However, diagnostic workup during admission does not always lead to effective diagnosis, often making these personalized treatment approaches challenging*.* Artificial intelligence is a robust area of research that is advancing diagnostic accuracy and aiding clinical decision making. With further iterations and implementation into clinical practice, this technology provides an efficient adjunct that can provide essential diagnostic and prognostic value in managing patients impacted by pathology of the heart-brain axis.

## Glossary

ACS: acute coronary syndrome
AF: atrial fibrillation
AHA: American Heart Association
AI: artificial intelligence
AIS: acute ischemic stroke
ANN: artificial neural network
ARIC: atherosclerosis risk in communities
AS: aortic stenosis
ASD: atrial septal defect
ASPECTS: Alberta stroke program computed tomography score
AUC: area under the curve
BNP: brain natriuretic peptide
CAD: coronary artery disease
CHD: congenital heart disease
CMR: cardiovascular resonance
CNS: central nervous system
CO: cardiac output
CS: cryptogenic stroke
CT: computed tomography
CTA: computed tomography angiography
CTP: computed tomography perfusion
CVA: cerebrovascular accident
DL: deep learning
DT: decision tree
EDV: end diastolic volume
EKG: electrocardiogram
EMR: electronic medical records
ESUS: embolic strokes of undetermined source
FCN: fully convolutional network
FDA: food and drug administration
FDG PET/CT: 18f-fluorodeoxyglucose PET/CT
FHS: Framingham heart study
GBM: gradient boosting machine
GLS: global longitudinal strain
HF: heart failure
HFrEF: heart failure with reduced ejection fraction
HPA: hypothalamic-pituitary-adrenal axis
ICAD: intracranial atherosclerotic disease
ICC: interclass correlation coefficients
IE: infective endocarditis
LA: left atrium
LR: logistic regression
LV: left ventricular
LVEF: left ventricular ejection fraction
LVO: large vessel occlusion
MACCEs: major adverse cardiac and cerebrovascular events
MCA: middle cerebral artery
MI: myocardial infarctions
ML: machine learning
MR: mitral regurgitation
MRI: magnetic resonance imaging
MRI-VPD: magnetic resonance plaque analysis system
NDAF: newly diagnosed atrial fibrillation
NLP: natural language processing
NSC: neurogenic stress cardiomyopathy
NSTEMI: non-ST elevation mi
PASCAL: PFO-associated stroke causal likelihood
PET: positron-emission tomography
PFO: patent foramen ovale
PISA: proximal isovelocity surface area
PPG: photoplethysmography
RE: relative speed error
RF: random forest
RMSE: root mean square error
RoPE: risk of paradoxical embolism
RSF: random survival forst
RV: right ventricular
SSIM: structural similarity index
STE: speckle-tracking echocardiography
STEMI: ST elevation mi
SVM: support vector machine
TCD: transcranial doppler ultrasound
TEE: transesophageal echocardiogram
TNN: transformer based neural networks
TOAST: trial of ORG 10172 in acute stroke treatment
TTC: takotsubo cardiomyopathy
TTE: transthoracic echocardiogram
VHD: valvular heart disease
